# Cancer screening prevalence and preference among hospitalized women with and without diabetes mellitus

**DOI:** 10.1371/journal.pone.0319681

**Published:** 2025-03-11

**Authors:** Margaret A. Mallari, Amteshwar Singh, Jocelyn Shubella, Waseem Khaliq

**Affiliations:** Department of Medicine, Division of Hospital Medicine, Johns Hopkins Bayview Medical Center, Johns Hopkins University School of Medicine, Baltimore, Maryland, United States of America; Washington University in St Louis, UNITED STATES OF AMERICA

## Abstract

**Objective:**

To determine the prevalence of nonadherence to breast cancer and colorectal cancer screening, associated risk factors, and screening preference among hospitalized women with and without diabetes aged 50–75 years who were cancer-free at baseline.

**Methods:**

A prospective study compared women with and without diabetes who were cancer-free (except for skin cancer) at baseline and between 50 and 75 years of age, admitted to the general medical service at an academic center were approached for study participation from December 1, 2014, to May 31, 2017. The study evaluated breast and colorectal cancer screening nonadherence prevalence, preference for screening locale, sociodemographic and clinical variables associated with nonadherence using multivariable logistic regression model.

**Results:**

Of 510 women, 39% had a prior diagnosis of diabetes mellitus, and 36% were African American. Women with diabetes were more likely to have obesity, reliance on assistive devices for ambulation, inability to work (have a disability), and a greater average number of comorbidities compared to women without diabetes. Women with or without diabetes were equally nonadherent with BRC (28% vs 36%, p = 0.6) and CRC (25% vs 28%, p = 0.51) screening guidelines. After adjustment for sociodemographic and clinical risk factors, only high risk for CRC (OR = 3.20, 95%CI; 1.03–9.91) was an independent risk factor associated with nonadherence to BRC among hospitalized women with diabetes. Whereas after similar adjustment, age younger than 60 years (OR = 2.91, 95%CI; 1.15–7.35) and current or prior smoking (OR = 2.80, 95%CI; 1.14–6.86) were associated with nonadherence to CRC among women with diabetes. 46% of women with diabetes expressed a preference for in-hospital screening for BRC, while 45% expressed a similar preference for CRC.

**Conclusion:**

Hospitalizations may offer additional screening opportunities as almost half of the women with diabetes preferred undergoing breast and colorectal cancer screening during a hospital stay.

## Introduction

Cancer is the second leading cause of death in the United States [1]. Cancer trends appear less favorable among women, compared to men [2]. Breast cancer (BRC) is the leading cancer among women, contributing to most new cancer cases per year in the United States and ranks second in terms of mortality among women [2]. Colorectal cancer (CRC) ranks third in cancer incidence and mortality among women. Both cancers share many similar risk factors, including age, obesity, physical activity status, genetic factors, smoking, and diabetes mellitus [3,4]. Women with diabetes mellitus are at a higher risk of cancer and cancer-related mortality [5,6].

Mammography and colonoscopy are effective evidence-based screening modalities for BRC and CRC, respectively, and have been shown to improve mortality. Despite the proven screening benefits of early detection and improved cancer-related mortality, women with diabetes mellitus are less likely to be screened for BRC/CRC as compared to their non-diabetic counterparts [7–9]. Patients with diabetes mellitus have poorer preventive care and suboptimal adherence to guideline-based screening for BRC/CRC due to co-morbidities [9]. This may, in part, be due to socioeconomic disadvantages, financial barriers, transportation issues, and logistical planning in the outpatient setting [9–14].

Women favor hospitalization time to undergo screening, which may represent a unique opportunity to capture the non-adherent population [15–17]. Therefore, we conducted this study to determine the breast and colorectal cancer screening nonadherence prevalence, associated risk factors, and screening preference among hospitalized women with and without diabetes aged 50-75 years who were cancer-free at baseline.

## Methods

### Study design and sample

Detailed enrollment methods have been published [18]. Briefly, we enrolled 510 women 50 to 75 years of age who were cancer-free (except for skin cancer excluding melanoma) at baseline and admitted to the general medical service at a major academic center from December 1, 2014, until May 31, 2017. Patients with multiple admissions during the study period were only offered enrollment on their first admission.

### Protocol and measures

Using a bedside survey, we collected information about sociodemographic, access to health care, BRC/CRC risk assessment, adherence to BRC/CRC screening, and medical comorbidities. Adherence to BRC/CRC was self-reported and defined according to the United States Preventive Services Task Force (USPSTF) [19,20].

Nonadherence with breast cancer screening recommendations was defined as having screening mammography more than 24 months before enrollment among women aged 52 or older in accordance with USPSTF guidelines [20]. Risk stratification for BRC was performed using the National Cancer Institute Breast Cancer Risk Tool [21]. Adherence to CRC screening was ascertained according to USPSTF recommendations; all individuals ages 50 to 75 years should undergo CRC screening via stool-based (Fecal Occult Blood test within one year) or direct visualization tests (flexible sigmoidoscopy within 5 years, or screening colonoscopy within 10 years) for CRC screening [19]. CRC risk stratification was assessed by inquiring about the personal history of Lynch syndrome, familial adenomatous polyposis (FAP), or inflammatory bowel disease (IBD).

Access to health care was ascertained with variables such as health insurance status and having a primary care physician. Disease burden was evaluated by assessing medical comorbidities, including those needed for the Charlson Comorbidity Index (CCI), a method of categorizing comorbidities by mortality risk status based on the International Classification of Diseases (ICD) diagnosis codes [22]. Socioeconomic information was evaluated by assessing demographic such as race, marital status, education, employment and annual household income.

At the end of the survey, the study coordinator provided the participants with one-on-one bedside education about CRC/BRC screening by sharing handouts with all study participants [23,24]. Upon completion of enrollment, each study participant was given a $10 gift card.

Six months after discharge from the hospital, study participants received a follow-up phone survey to determine adherence to BRC/CRC screening guidelines. This short follow-up survey was conducted to determine whether these women visited primary care providers after hospital discharge to discuss BRC/CRC screening and if they received BRC/CRC screenings post-hospitalization. The Institutional Review Board at the academic medical center approved the study protocol. All study participants provided their written informed consent for participation.

### Outcome and evaluation

The primary outcome was the prevalence of nonadherence to BRC/CRC screening guidelines among hospitalized women with and without diabetes and sociodemographic and clinical variables associated with nonadherence to BRC/CRC screening among hospitalized women with and without diabetes. We also evaluated preference toward cancer screening tests locale among these hospitalized women stratified by diabetes status as a secondary outcome.

### Statistical methods

We calculated the study sample using a 5% margin of error with a confidence level of 95% and a response distribution of 50% (http://www.raosoft.com/samplesize.html). Respondent characteristics are presented as proportions and means. We used unpaired t-test and Chi-square test to compare demographic and socioeconomic characteristics between women with and without diabetes mellitus. T-tests and Chi-square tests determined significance at a p-value ≤  0.05. We used multivariable logistic regression models for analyses to predict odds ratios (OR) of nonadherence with BRC and CRC screening. Logistic regression models were adjusted for a priori sociodemographic and clinical comorbidity risk factors thought to influence screening adherence. The data were analyzed using Stata, Version 13.1.

## Results

Among the 510 hospitalized women participants, 39% had a prior diagnosis of diabetes mellitus. The mean age of participants was 60.5 years (SD =  6.9). Table 1 describes the characteristics of study participants by diabetes status. Hospitalized women with diabetes were more likely to have obesity, ambulation assistive-device dependence (ambulate using a cane, walker, or wheelchair), bedbound status, and were likely on disability or unable to work. Women with diabetes had more medical comorbidities (age adjusted CCI > 3) than their non-diabetic counterparts. However, they were less likely to use alcohol (Table 1).

**Table 1:** Characteristics of study population.

Characteristics ^Ŧ^	Patients without Diabetes Mellitus (N = 312)	Patients with Diabetes Mellitus (N = 198)	p-value*
Age in years, mean (SD)	60.1 (7)	61.1 (6.7)	0.11
Race			0.15
White, n (%)	195 (63)	114 (58)	
African American, n (%)	104 (33)	80 (40)	
Others, n (%)	13 (4)	4 (2)	
Married or living with a partner, n (%)	97 (31)	58 (29)	0.76
High school or more years of education, n (%)	256 (82)	150 (76)	0.09
Employment status, n (%)			**<0.001**
Employed	86 (28)	27 (13)	
Unemployed	23 (7)	11 (6)	
Retired	82 (26)	59 (30)	
Disability/unable to work	121 (39)	101 (51)	
Ambulatory function			**0.02**
Ambulate without assistance, n (%)	216 (69)	95 (48)	
Ambulate with cane or walker, n (%)	84 (27)	90 (45)	
Chronic disable, wheelchair or bed bound n (%)	12 (4)	13 (7)	
Annual household income < $20,000, n (%)	129 (42)	96 (49)	0.13
Uninsured, n (%)	4 (1)	1 (0.5)	0.08
No primary care physician, n (%)	34 (11)	12 (6)	0.08
Admitted as observation, n (%)	21 (7)	8 (4)	0.24
Principle diagnosis by system at admission, n (%)			0.20
General internal medicine	108 (34)	53 (27)	
Cardiovascular	40 (13)	38 (19)	
Pulmonary	58 (18)	36 (18)	
Gastrointestinal	43 (14)	22 (11)	
Neurology	3 (1)	4 (2)	
Nephrology	19 (6)	15 (8)	
Oncology	6 (2)	2 (1)	
Rheumatology	12 (4)	6 (3)	
Psychiatry	3 (1)	1 (0)	
Infectious disease	12 (4)	13 (7)	
Others	8 (3)	8 (4)	
Length of stay in days, mean (SD)	4.5 (5.7)	5.5 (4.2)	**0.03**
Current Smoker, n (%)	108 (35)	54 (27)	0.08
Alcohol use, n (%)	102 (33)	30 (15)	**<0.001**
Age adjusted Charlson Comorbidity Index (CCI) > 3, ^¥^ n (%)	69 (22)	138 (70)	**<0.001**
Body Mass Index (BMI) kg/m^2^, n (%)			**<0.001**
Less than 25	89 (29)	23 (11)	
25-29.9	51 (16)	35 (18)	
≥30	172 (55)	140 (71)	

^Ŧ^For some patients, the variables had missing value.

* Chi-Square, Fisher’s exact statistic (where at least 20% of frequencies were < 5), and Unpaired t-test statistic.

¥ Charlson comorbidity index (CCI) -- scores of 0, 1, 2, and 3 predicting 10-year survival rates of 93%, 73%, 52%, and 45%, respectively

Table 2 shows the prevalence of BRC/CRC screening adherence, risk stratification, and patients’ preference for screening mammography and colonoscopies, respectively. Among hospitalized women with diabetes, 28% were nonadherent to screening mammography, and 25% were nonadherent to colorectal cancer screening. 29% never had a screening colonoscopy, whereas 5% never had a screening mammography. There were no statistical differences in the prevalence of nonadherence to either BRC or CRC screening guidelines between hospitalized women with and without diabetes. Additionally, the two groups had no differences in family history or high risk for BRC or CRC. Upon exploring preference for a clinical locale for BRC/CRC screenings, almost half of the hospitalized women with diabetes preferred getting screening mammograms and colonoscopies (46%, p-value 0.62, and 45% p-value 0.23, respectively) if due and offered during hospitalization (Table 2).

**Table 2:** Cancer screening prevalence and preferences of study women while hospitalized.

Screening adherence and preference for screening ^Ŧ^	Patients without Diabetes Mellitus (N = 312)	Patients with Diabetes Mellitus (N = 198)	p-value*
Breast cancer risk, adherence, and preference			
Nonadherent to screening mammography, n (%)	113 (36)	56 (28)	0.60
Never had screening mammogram, n (%)	30 (10)	9 (5)	**0.04**
Family history of breast cancer, ^†^ n (%)	54 (17)	35 (18)	0.91
5-year-risk prediction ≥ 1.7% using Gail model, ^¥^ n (%)	126 (40)	84 (42)	0.65
Preference of screening mammogram location assuming can be done anywhere, n (%)			
Inpatient setting during hospitalization	156 (50)	91 (46)	0.62
Outpatient mammography center	86 (28)	52 (26)	
Will not do either setting	23 (7)	19 (10)	
Either setting will be fine	47 (15)	36 (18)	
Colorectal cancer risk, adherence, and preference			
Nonadherent to colorectal cancer screening, n (%)	87 (28)	50 (25)	0.51
Never had screening colonoscopy, n (%)	116 (37)	57 (29)	**0.05**
Family history of colorectal cancer, ^††^ n (%)	36 (12)	22 (11)	1.00
High risk for colorectal cancer, ^¥¥^ n (%)	27 (9)	18 (9)	0.87
Preference of screening colonoscopy location assuming can be done anywhere, n (%)			0.23
Inpatient setting during hospitalization	166 (53)	88 (45)	
Outpatient clinic colonoscopy	73 (23)	55 (28)	
Will not do either setting	24 (8)	15 (7)	
Either setting will be fine	49 (16)	39 (20)	

^Ŧ^For some patients the variables had missing value.

*Chi-Square, (Yates corrected p value where at least 20% of frequencies were < 5) and Unpaired t-test statistic.

^†^Family History of Breast cancer was defined as breast cancer in first-degree relatives like mother, sisters, or daughters.

^¥^Gail score was estimated using the National Cancer Institute Breast Cancer Risk Tool (http://www.cancer.gov/bcrisktool/).

^††^Family history of colorectal cancer was defined as colorectal cancer in first-degree relatives like father, mother, brother, sisters, son, or daughters.

^¥¥^ History of Lynch syndrome, familial adenomatous polyposis, or inflammatory bowel disease

We used multivariate logistic regression modeling to evaluate sociodemographic and clinical variables associated with BRC/CRC nonadherence among hospitalized women with diabetes (n = 198). After adjustment of sociodemographic and clinical variables among women with diabetes, the adjusted odds of nonadherence to BRC screening was 3.20 times higher (adjusted OR: 3.20; 95% CI 1.03–9.91) among diabetic hospitalized women who were at high risk for developing CRC as compared to diabetic women at average risk for developing CRC (Table 3). Similarly, adjusted odds of nonadherence to CRC screening were 2.91 times higher (adjusted OR: 2.91; 95% CI 1.15–7.35) in younger women (age less than 60 years) and 2.80 times higher (adjusted OR: 2.80; 95% CI 1.14–6.86) among current smokers or former smokers compared to diabetic women age ≥  60 years, and never smoker respectively (Table 3).

**Table 3:** Predictors of nonadherence to breast and colorectal cancer screening recommendations among hospitalized women with diabetes mellitus only (n = 198).

Nonadherence Risk factors	Odds Ratio (95% CI)	
Predictor of nonadherence to Breast cancer screening	Unadjusted Model ^*^	Adjusted Model ^**^
Age < 60 years	**2.26 (1.20 – 4.24)**	2.40 (0.99 – 5.76)
African American and other races (versus White)	0.91 (0.49 – 1.71)	1.04 (0.49 – 2.22)
Married or living with a partner	1.34 (0.70 – 2.61)	1.89 (0.82 – 4.36)
Education less than High school	1.98 (0.99 – 3.94)	1.70 (0.75 – 3.80)
Unemployed/retired/disabled	**3.62 (1.05 – 12.56)**	3.87 (0.96 – 15.6)
Annual household income less than $20,000	1.65 (0.88 – 3.11)	1.24 (0.56 – 2.76)
No primary care provider	4.99 (2.09 – 11.9)	1.04 (0.24 – 4.62)
Current and ex-smoker (versus never smoker)	1.47 (0.77 – 2.84)	1.10 (0.51 – 2.38)
Ambulatory status (walk with cane or bedbound)	1.31 (0.70 – 2.45)	1.27 (0.62 – 2.60)
Alcohol use	0.90 (0.38 – 2.16)	0.85 (0.31 – 2.29)
Nonadherence to colorectal cancer screening	**2.42 (1.22 – 4.78)**	2.10 (0.94 – 4.69)
High risk for colorectal cancer	2.18 (0.81 – 5.86)	**3.20 (1.03 – 9.91)**
Family history of colorectal cancer	1.18 (0.45 – 3.07)	1.40 (0.48 – 4.10)
High risk for breast cancer	0.91 (0.49 – 1.71)	1.53 (0.62 – 3.77)
Family history of breast cancer	1.00 (0.45 – 2.27)	0.78 (0.26 – 2.31)
Obesity (BMI ≥ 30 kg/m^2^)	1.20 (0.60 – 2.39)	1.20 (0.53 – 2.70)
Age adjusted CCI > 3	0.64 (0.33 – 1.23)	0.71 (0.28 – 1.59)
*Predictor of nonadherence to colorectal cancer screening*	**Unadjusted Model** ^*^	**Adjusted Model** ^***^
Age < 60 years	**3.19 (1.64 – 6.22)**	**2.91 (1.15 – 7.35)**
African American and other races (versus White)	1.51 (0.79 – 2.87)	1.50 (0.68 – 3.34)
Married or living with a partner	1.19 (0.59 – 2.38)	1.71 (0.71 – 4.10)
Education less than High school	1.71 (0.84 – 3.47)	1.15 (0.49 – 2.71)
Unemployed/retired/disabled	1.21 (0.46 – 3.20)	1.17 (0.35 – 3.87)
Annual household income less than $20,000	1.12 (0.58 – 2.13)	1.41 (0.59 – 3.34)
No primary care provider	1.52 (0.44 – 5.29)	1.22 (0.24 – 6.23)
Current and ex-smoker (versus never smoker)	**2.41 (1.17 – 4.99)**	**2.80 (1.14 – 6.86)**
Ambulatory status (walk with cane or bedbound)	0.90 (0.47 – 1.70)	1.10 (0.50 – 2.37)
Alcohol use	1.10 (0.45 – 2.63)	0.66 (0.23 – 1.93)
Nonadherence to breast cancer screening	**2.42 (1.22 – 4.78)**	2.19 (0.99 – 4.85)
High risk for breast cancer	**0.38 (0.19 – 0.77)**	0.36 (0.12 – 1.10)
Family history of breast cancer	0.70 (0.28 – 1.71)	1.54 (0.41 – 5.75)
High risk for colorectal cancer	0.34 (0.08 – 1.55)	0.31 (0.06 – 1.76)
Family history of colorectal cancer	0.44 (0.12 – 1.55)	0.43 (0.11 – 1.76)
Obesity (BMI ≥ 30 kg/m^2^)	0.74 (0.37 – 1.48)	0.51 (0.22 – 1.20)
Age adjusted CCI > 3	0.90 (0.45 – 1.79)	1.75 (0.70 – 4.36)

*Unadjusted model.

**Adjusted model for cancer screening related medical risk factors thought to potentially influence the adherence to breast cancer screening among hospitalized women (age, race, marital status, education, employment status, annual household income, having no primary care provider, smoking status, ambulatory status, alcohol use, nonadherence to colorectal cancer screening, high risk for colorectal cancer, family history for colorectal cancer, high risk for developing breast cancer, family history for breast cancer, obesity, and age-adjusted Charlson comorbidity index (CCI).

***Adjusted model for cancer screening related medical risk factors thought to potentially influence the adherence to colorectal cancer screening among hospitalized women (age, race, marital status, education, employment status, annual household income, having no primary care provider, smoking status, ambulatory status, alcohol use, nonadherence to breast cancer screening, high risk for developing breast cancer, family history for breast cancer, high risk for colorectal cancer, family history for colorectal cancer, obesity, and age-adjusted Charlson comorbidity index (CCI).

Appendix A provides the predictors for BRC and CRC nonadherence among study population without diabetes for reference comparison.

## Appendix A

### Predictors of nonadherence to breast and colorectal cancer screening recommendations among hospitalized women without diabetes

**Table:** 

Nonadherence Risk factors		Adjusted Odds Ratio (95% CI)
Predictor of nonadherence to Breast cancer screening		Adjusted Model ^*^
Age < 60 years		0.65 (0.34 – 1.28)
African American and other races (versus White)		0.58 (0.32 – 1.04)
Married or living with a partner		0.77 (0.40 – 1.46)
Education less than High school		0.57 (0.27 – 1.21)
Unemployed/retired/disabled		1.08 (0.53 – 2.18)
Annual household income less than $20,000		1.20 (0.63 – 2.27)
No primary care provider		**7.60 (2.93 – 19.7)**
Current and ex-smoker (versus never smoker)		1.64 (0.89 – 2.99)
Ambulatory status (walk with cane or bedbound)		1.37 (0.74 – 2.56)
Alcohol use		1.02 (0.56 – 1.87)
Nonadherence to colorectal cancer screening		**5.44 (2.91 – 10.2)**
High risk for colorectal cancer		0.76 (0.26 – 2.24)
Family history of colorectal cancer		0.78 (0.32 – 1.88)
High risk for breast cancer		0.58 (0.27 – 1.23)
Family history of breast cancer		0.71 (0.28 – 1.81)
Obesity (BMI ≥ 30 kg/m^2^)		1.08 (0.62 – 1.90)
Age adjusted CCI > 3		1.17 (0.57 – 2.42)
*Predictor of nonadherence to colorectal cancer screening*		**Adjusted Model** ^**^
Age < 60 years		**2.64 (1.29 – 5.39)**
African American and other races (versus White)		0.95 (0.51 – 1.76)
Married or living with a partner		0.99 (0.50 – 1.94)
Education less than High school		1.55 (0.74 – 3.21)
Unemployed/retired/disabled		0.82 (0.38 – 1.74)
Annual household income less than $20,000		1.58 (0.81 – 3.11)
No primary care provider		0.96 (0.40 – 2.34)
Current and ex-smoker (versus never smoker)		0.69 (0.36 – 1.30)
Ambulatory status (walk with cane or bedbound)		1.35 (0.69 – 2.64)
Alcohol use		0.59 (0.31 – 1.14)
Nonadherence to breast cancer screening		**5.52 (2.96 – 10.3)**
High risk for breast cancer		0.78 (0.34 – 1.79)
Family history of breast cancer		1.39 (0.51 – 3.78)
High risk for colorectal cancer		0.12 (0.02 – 1.00)
Family history of colorectal cancer		1.61 (0.66 – 3.92)
Obesity (BMI ≥ 30 kg/m^2^)		1.04 (0.58 – 1.88)
Age adjusted CCI > 3		0.48 (0.20 – 1.14)

The post-hospitalization follow-up survey had a low overall response rate of 24% (n = 123). The response rate was slightly higher among women with diabetes at 25% (n = 50) compared to 23% (n = 73) among women without diabetes. Among respondents, 96% (n = 48) of women with diabetes followed up with their primary care provider, compared to 94% (n = 68) of women without diabetes. Regarding screening discussions, 79% (n = 38) of women with diabetes discussed BRC screening and 71% (n = 34) discussed CRC screening with their primary care provider. In contrast, only 57% (n = 83) and 53% (n = 77) of women without diabetes discussed BRC and CRC screening, respectively. Readmission rates were similar, with 54% (n = 27) of women with diabetes reporting readmission compared to 57% (n = 41) of women without diabetes. Post-hospitalization, 62% (n = 31) of women with diabetes received BRC screening, and 38% (n = 19) received CRC screening. Among women without diabetes, 62% (n = 45) underwent BRC screening, and 37% (n = 27) received CRC screening. For women with diabetes who were nonadherent to BRC screening, only 16% (n = 5) received BRC screening post-hospitalization, compared to 84% (n = 26) of diabetic women who were adherent. Similarly, only 11% (n = 2) of women with diabetes who were nonadherent to CRC screening received CRC screening post-hospitalization, compared to 89% (n = 17) of diabetic women who were adherent. None of these differences were statistically significant in the final analysis.

## Discussion

This prospective intervention study demonstrates that cancer-free hospitalized women with and without diabetes have alarmingly low rates of self-reported adherence to breast and colorectal cancer at baseline. Almost half of these women report a preference for in-hospital screening, which may be an impactful intervention to improve cancer screening adherence.

Studies have previously shown that women with diabetes have lower BRC and CRC screening rates compared to those without diabetes [8,9]. Our prospective survey-based study negates these findings, although a quarter of women with diabetes reported nonadherence to BRC and CRC cancer screening, (28% and 25% respectively). Lack of appropriate age-related cancer screening in nonadherent women with diabetes poses a population health risk of increased morbidity and mortality, as well as higher healthcare costs.

Our study reports that age < 60 years is associated with lower adherence to BRC/CRC screening, which has been reported previously in African American, Korean-American, and Korean immigrant women regardless of the diabetes status [25]. Younger age may be associated with low adherence rates due to low linkage to outpatient care [26,27]. Smoking (current or past) and nonadherence to BRC screening were also found to be predictors of nonadherence to CRC screening amongst the participants. Smoking status has previously been found to be associated with low likelihood of adherence with breast, endometrial, and colorectal cancer screening in the outpatient setting. Our study found similar results in the inpatient setting for colorectal cancer screening only. Smoking status was not associated with non-adherence to breast cancer screening in our study [28]. Concerted efforts such as smoking cessation programs, mailed/telephonic outreach [29], accessible outpatient appointments [30], and offering inpatient screening interventions for at-risk nonadherent patients may be of value in improving adherence. Although we were unable to find a reasonable explanation for these findings, we suspect that women at high risk of BRC could be more cautious about CRC and perceive cancer risk differently when compared to women at high risk of CRC, primarily due to a lack of awareness of risk. This may be partly due to the lack of a standardized CRC risk stratification tool or the underutilization of comprehensive CRC risk assessment in the outpatient setting (when compared to BRC risk stratification). Also, notably, the societal awareness of BRC screening may be higher due to advertisements, campaigns, etc.

Patients with diabetes often have multiple comorbidities and poor ambulatory capacity, which may make it more difficult for them to navigate outpatient screening interventions in addition to scheduling and transportation challenges. Although our study doesn’t show these factors as predictors of self-reported nonadherence to screening, about half of the women preferred in-hospital screening. This preference may be related to convenience [30]. Post-discharge outpatient adherence was reported to be relatively low for both BRC and CRC, despite more than 90% of women reporting interest in cancer screening in either inpatient or outpatient settings. These findings should prompt healthcare organizations and insurance companies to examine further the benefits and cost-effectiveness of inpatient BRC and CRC screening for select at-risk women with diabetes. Such patient-centered initiatives would help improve screening adherence.

Several limitations of this study should be considered. First, the study was conducted at a single hospital, limiting the findings’ generalizability. Second, the self-reporting methodology has reliability limitations inherent to the survey-based design. Third, our study only included women as part of our study. Breast cancer is the most commonly diagnosed cancer among women [2]. Additionally, female sex is known to be associated with non-compliance with CRC screening as women perceive health and risk of developing CRC differently from men [31,32]. Thus, evaluating prevalence, preferences for screening, and predictors for BRC/CRC nonadherence for women with diabetes should be considered a strength as it highlights gender-specific preferences and barriers to screening. Lastly, the number of patients who responded to the follow-up survey was small, a common problem encountered in many other studies evaluating screening preference and barriers among hospitalized women [32,33]. Nevertheless, the study provides some insight into the pattern of nonadherence to BRC/CRC cancer screening among hospitalized women with diabetes during hospitalization and after discharge from the hospital.

### Implementation

Hospitalization is a significant event in patients’ lives as they reflect on their overall health in general, including preventive care options. Discussion of cancer risk and recommended screening interventions that align with patient preferences may help improve adherence. Healthcare organizations should create an environment where system-based barriers can be minimized so that hospital providers can counsel about cancer screening and high-risk patients can undergo screening tests regardless of clinical locale

## Conclusion

Our study reports that a quarter of cancer-free hospitalized women with and without diabetes are nonadherent to breast and colorectal cancers screening and almost half of these women report a preference for in-hospital screening. Future outcomes research is needed to determine the feasibility and cost-effectiveness of cancer screening in hospital settings.
